# Sub-optimal nutrient regime coupled with *Bacillus* and *Pseudomonas* sp. inoculation influences trichome density and cannabinoid profiles in drug-type *Cannabis sativa*

**DOI:** 10.3389/fpls.2023.1131346

**Published:** 2023-05-19

**Authors:** Cailun A. S. Tanney, Dongmei Lyu, Timothy Schwinghamer, Anja Geitmann, Eric D. Ruan, Donald L. Smith

**Affiliations:** ^1^ Department of Plant Science, McGill University, Sainte-Anne-de-Bellevue, QC, Canada; ^2^ Lethbridge Research and Development Centre, Agriculture and Agri-food Canada, Lethbridge, AB, Canada; ^3^ School of Chemistry and Chemical Engineering, Sichuan Institute of Arts and Science, Dazhou, Sichuan, China

**Keywords:** cannabis, trichomes, nutrients, microbes, inflorescences, inoculation, cannabinoids, stereomicroscopy

## Abstract

*Cannabis sativa* remains under heavy legal restriction around the globe that prevents extensive investigations into agricultural applications for improving its development. This work investigates the potential of specific plant growth-promoting rhizobacteria (PGPR) to improve *Cannabis* cannabinoid yield through increased trichome densities on floral organs, and to determine if sub-optimal environmental conditions would affect the outcomes of PGPR presence by altering plant development and cannabinoid profiles. Here, *Pseudomonas* sp. or *Bacillus* sp. were applied to the root system either separately or in a consortium to determine the effect of this bacterial treatment on the density of stalked glandular trichomes. Further, a low nutrient regime was applied for the first half of plant development to determine if an environmental stressor interacts with the effects of the microbial treatments on stalked trichome densities. Following 8 weeks of flower development, trichome density on calyces and bracts of inflorescences were determined using microscopy. Our findings unexpectedly indicate that recommended nutrient levels were linked to a decreasing trend in trichome densities with PGPR inoculations, but a low nutrient regime coupled with PGPR treatment increased them. Cannabinoid content is partially consistent with these results, in that a low nutrient regime increased the abundance of key cannabinoids compared to recommended regimes, with *Bacillus* sp. inoculation linked to the greatest number of significant changes between the two nutrient regimes. Overall, this work provides insight into how PGPR presence affects *Cannabis* stalked trichome development and cannabinoid profiles, and how environmental stressors can affect, and even enhance, trichome densities and influence major cannabinoid production, thereby pointing towards avenues for reducing the reliance on synthetic fertilizers during plant production without compromising yield.

## 1 Introduction

With Canada having set the precedent for nation-wide recreational *Cannabis* (hereafter, cannabis) legalization in North America, and Malta now the first European Union country to legalize it for personal use in 2021 (Authority on the Responsible Use of Cannabis Act, 2021), global demand for cannabis products is expected to rise sharply as more countries follow suit. Research on cannabis has slowly begun to catch up to the progress made in the agricultural science boom of the 20^th^ century, but research remains primarily focused on medicinal and analytical aspects. This is largely due to accessibility, as despite increasing legalization, the regulations governing cannabis cultivation for agricultural research remain challenging. While a highly profitable industry, with heavy legislation limiting product types and consumer availability, research focused on agricultural applications remains scarce. Projected to reach 102.2 billion USD by 2030 in the global legal market (Grand View Research, 2022), it is now time that validations of modern agricultural methods be carried out for cannabis to ensure this expanding industry benefit from research-backed practices.

Female cannabis inflorescences are the primary source of cannabinoids and terpenes (Ohlsson et al., 1971), with stalked glandular trichomes on the surface of floral organs and bracts being the key secretory structures (Mahlberg and Eun, 2004; Livingston et al., 2020). Recent work has used a deep learning pipeline to identify stages of trichome development based on their age-based transition through clear-milky-brown phenotypes, providing a sophisticated tool for cannabis product investigation (Sutton et al., 2023). As these trichomes are the source of the bulk of cannabinoids in cannabis products, it is imperative that research aimed at improving cannabinoid yield be directed towards these structures.

Efforts at manipulating cannabis yields are largely focused on abiotic environmental conditions for plant culture (reviewed by Backer et al., 2019; Jin et al., 2019; Desaulniers Brousseau et al., 2021) or on post-harvest processing (reviewed by Addo et al., 2021). Variables characterizing indoor growing conditions are being investigated for their potential to affect metabolite profiles and yields in a cultivar-specific manner. Efforts are typically directed toward lighting systems, in the context of light spectrum and intensities (Hawley et al., 2018; Magagnini et al., 2018; Danziger and Bernstein, 2021; Rodriguez-Morrison et al., 2021; Wei et al., 2021; Westmoreland et al., 2021) and fertilizer nutrient applications (Caplan et al., 2017; Bernstein et al., 2019; Yep and Zheng, 2020, Bevan et al., 2021). The concept of optimizing nutrient applications for cannabis cultivation by precise manipulation of individual compounds is beginning to attract research interest. Bevan et al. (2021) demonstrated how nitrogen, phosphorus, and potassium contents can impact inflorescence yield in drug-type cannabis. They pointedly identified how potassium had no bearing on yield, suggesting that the administration of this nutrient is likely provided in excess and may be a resource drain. While this conclusion was tested only in soilless environments, it is a step towards optimizing grow operations and opens avenues toward tailoring production protocols. Research is warranted on the potential of the use of microbial supplements to improve cannabis yield.

The potential of manipulating the phytomicrobiome to optimize cannabis yield is in its infancy. Plant growth-promoting rhizobacteria (PGPR) are well-established fertilizer supplements for other crop species. They are known to support plant development through improving water and nutrient acquisition and establishing synergistic relationships with their plant hosts through the production of phytohormones (Berendsen et al., 2012; Sivasakthi et al., 2014; Kundan et al., 2015) or signal compounds (Backer et al., 2019; Lyu et al., 2022a). Recent work has demonstrated that the administration of PGPR has potential applications for improving cannabis development and metabolic yields, but details are lacking. Lyu et al. (2022a) demonstrated an improvement of inflorescence fresh weight with three separate rhizobacterial species, of which two increased the number of inflorescences per plant. Using hemp cultivars, Pagnani et al. (2018) revealed that PGPR inocula also affected the metabolite profiles of their cultivars. With regards to pathogen control, Balthazar et al. (2022a) have recently shown the efficacy of twelve strains of *Bacillus* and *Pseudomonas* against culturable cannabis fungal pathogens and found 5 strains had a significant biocontrol impact on reducing gray mold development in planta. Going further, they also confirmed that there were no virulence or toxin factor genes in the genome of the favourable potential strains (Balthazar et al., 2022a). These findings further support the use of beneficial microbes for sustainable cannabis yield improvement but through pathogen control; a concept further explored in Balthazar et al. (2022b) with a focus on *Pseudomonas* sp. applications.

In addition to whole-plant effects, it has been demonstrated in other plant species that PGPR can help increase the content of essential oils, which in turn have been linked to increases in trichome counts (Copetta et al., 2006; Banchio et al., 2008). More specifically, an environmental stressor has been found to increase essential oil yields in *Melissa officinalis* (Eshaghi Gorgi et al., 2022), and in *Cymbopogon citratus* this has further been linked to increases in trichome counts (Mirzaie et al., 2020). With the link between PGPR, essential oil production, and trichome development established for other crops, we wanted to assess whether similar relationships could be detected in cannabis. To determine if PGPR can influence cannabis stalked trichome densities, and if an environmental stressor can amplify these results, we inoculated cannabis plants with two PGPR strains, separately and in a consortium. In addition, a low nutrient regime for the first 6 weeks of development was applied to determine if an environmental stressor enhances any PGPR effects with regards to trichome development.

## 2 Materials and methods

### 2.1 Plant propagation and maintenance

Female *Cannabis sativa* L. plants of the cannabis variety “CBD Kush” were grown from cuttings sourced from in-house mother plants at Macdonald Campus, Saint-Anne-de-Bellevue, Quebec, in a Canada Revenue Agency and Health Canada approved research laboratory (license no. LIC-5AZZW7S4GM-2019). Mother plants were inspected for any signs of nutrient deficiency, pathogens, or pest damage. Medium-thick branches (~2 mm diameter) were cut and placed in water to prevent wilting. All leaves, with the exception of the top three fully-formed leaves, were removed from the stem and the outermost halves of the remaining leaves were clipped off. The ends of cuttings were trimmed to a 45° angle and dipped in Stim Root No. 2 powder (Master Plant-Prod Inc., Brampton, ON, Canada), after which they were placed in 3 cm pre-soaked rockwool cubes (Grodan, Roermond, Netherlands) on mesh trays (53 × 27 × 6 cm, Bootstrap Farmer, Downington, PA, USA) inside propagation trays (54 × 28 × 6 cm, Mondi, Vancouver, BC, Canada). Two L of VeloKelp nutrient solution (pH 5.6, Remo Nutrients; Remo Brands Inc., Maple Ridge, BC, Canada) at Transplant concentration (**Tables 1**, **2**) were poured into the trays and replaced once per week. Prepared trays were covered with a vented mini greenhouse (54 × 28 × 19 cm; Mondi, Vancouver, BC, Canada) and placed on a propagation rack for three to four weeks, until sufficient roots were observed (conditions – light at approximately 150 µmol m^-2^ s^-1^, 24 h photoperiod, 75-95% humidity, 24-25°C).

**Table 1:** Nutrient application schedule for inoculated cannabis cv. CBD Kush.

	Week												
	Transplant	1	2	3	4	5	6	7	8	9	10	11	12
**Average Daily Water Quantity (mL)**	250	150	150	150	150	250	250	250	250	250	250	250	250
**Low Nutrients**	1.3 mL L^-1^ VeloKelp	1.84 mL L^-1^ of VeloKelp						2.2 mL L^-1^ each of VeloKelp, Micro, MagNifiCal, Bloom, Astroflower					Water
**Recommended Nutrients**	1.3 mL L^-1^ VeloKelp	1.84 mL L^-1^ each of VeloKelp, Micro, Grow, MagNifiCal				2.2 mL L^-1^ each of VeloKelp, Micro, MagNifiCal, Bloom, Astroflower							Water

Under each nutrient regime, three series of healthy cuttings were transplanted into 15 cm pots (Teris, Laval, QC, Canada) containing pre-soaked Agromix G6 soil (300 mL of water per 400 g; Farfad Inc., Saint-Bonaventure, QC, Canada) and grown under vegetative conditions (approximately 150 µmol m^-2^ s^-1^, 18 h photoperiod, 20-22°C, 65% relative humidity) for four weeks. Vegetative plants were given the recommended nutrient regime of 150 mL water and nutrient application according to week of vegetative growth (**Table 1**) as per manufacturer guidelines and Lyu et al. (2022a) (Nutrients: MagNifiCal, Micro, VeloKelp, Grow at pH 6.3, Remo Nutrients). Following this period, plants were transferred to flowering conditions (approximately 150 µmol m^-2^ s^-1^, 12 h photoperiod, 20-22°C, 65% relative humidity) and given a regime of 250 mL water and nutrient application according to the week of flowering growth (**Table 1**) as per manufacturer guidelines and Lyu et al. (2022a) (Nutrients: MagNifiCal, Micro, VeloKelp, Astroflower, Bloom at pH 6.3, Remo Nutrients); only water was given in the final week of development, as per guidelines. Plants were grown under flowering conditions for a total of 8 weeks. Plants undergoing the low nutrient regime were given the same volume of nutrient-containing solution under the same growing conditions as the recommended nutrient regime. However, from Week 1 through 6, three of the four nutrient mixes were omitted and only the VeloKelp nutrient was provided at the same concentration as when combined with other nutrients as part of the recommended regime (pH 6.3, Remo Nutrients), creating a nutrient deficiency (**Table 1**). Following Week 6, complete nutrients, as described above, were given for a total of 5 weeks until Week 12, during which only water was provided (**Table 1**). **Table 2** provides the NPK content of each individual nutrient. Under low nutrients, series were started on 19 Apr 2021, 13 Jan 2022, and 24 Mar 2022 between 09h00-11h00. Under recommended nutrients, series were started on 19 Jun 2021, 18 Dec 2021, 13 Jan 2022 between 09h00-11h00. Daily plant care and maintenance occurred between 09h00–12h00.

**Table 2:** NPK content of the nutrient solutions applied to cannabis cv. CBD Kush.

	NPK Content per Nutrient Solution					
	VeloKelp	Micro	Grow	MagNifiCal	Bloom	Astroflower
**Nitrogen**	1%	1%	2%	3%	1%	1%
**Phosphorous**	1%	0%	3%	0%	4%	6%
**Potassium**	1%	1%	5%	0%	7%	11%

### 2.2 Bacterial inoculum preparation and delivery

*Pseudomonas* sp. (*Pseudomonas koreensis*, AF468452) and *Bacillus* sp. (*Bacillus mobilis*, KJ812449), originally isolated and identified by Fan et al. (2020) and previously studied with cannabis applications in Lyu et al. (2022a), were stored at -80 °C in glycerol and were revived by streaking onto petri plates containing sterile (30 min, 121°C) King’s Medium B (KB; 20.0 g L^-1^ protease peptone, 1.5 g L^-1^ K_2_HPO_4_, 10.0 g L^-1^ glycerol, 0.25 g L^-1^ MgSO_4_•7 H_2_O) and incubating at 28 °C overnight. Bacterial suspensions were prepared by scraping colonies from the plate surface into a beaker containing approximately 75 mL of sterile liquid KB medium and grown overnight at 28°C, rotating at 150 rpm. The following day, 30 mL of the inoculated media was distributed into 50 mL falcon tubes. Tubes were centrifuged at 1,000 rpm for 10 min (Sorvall Biofuge Pico, Kendro Laboratory Products, Asheville, NC, USA). The supernatant was discarded, and pellets were washed in 10 mL of 10 mM MgSO_4_. Following the wash, resuspended pellets were diluted to 0.1 OD at 600 nm (Ultraspec 4050 Pro UV/Visible spectrophotometer), using 10 mM MgSO_4_ as the blank.

Prepared inoculations were dispensed onto the soil immediately surrounding the base of the transplanted cuttings, which remained in the rockwool cubes when moved to soil, using a serological pipette on the day of transplantation. Ten mL of each inoculum was dispensed onto each cutting, and for the consortium treatment five mL of each inoculum component was dispensed. Control treatments received 10 mL of 10 mM MgSO_4_. Four plants per inoculation treatment group were prepared per nutrient regime experimental series and organized in a randomized complete block design. Four blocks per experimental series were created, with 3 experimental series per nutrient regime grown, providing a total of 12 true replicates.

### 2.3 Quantification of trichome density

**Figure 1:**
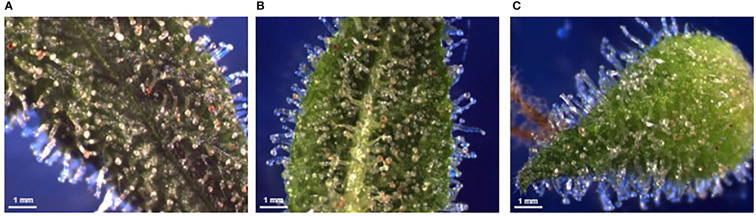
Examples of stereomicrographs used for determination of trichome density. Bract adaxial epidermis **(A)**, bract abaxial epidermis **(B)**, and calyx epidermis **(C)**. Visible tissue area was selected and stalked trichomes were manually counted.

To maintain consistency when sampling and to account for differences in plant height, one inflorescence per plant was removed at 3-5 nodes down from the apex inflorescence. Inflorescences were chosen based on size to ensure enough organ tissue would be available from a single inflorescence for dissection. Inflorescences were dissected down to individual calyces and bracts using razor blades, forceps, tweezers, and dissecting scissors. A minimum of six calyces and eight bracts were isolated, of which four calyces, four bract abaxial epidermis, and four bract adaxial epidermis surfaces were imaged (**Figure 1**), resulting in a total of twelve images per inflorescence, per plant in each inoculation treatment group for all experimental series. Tissues were imaged on a clear petri dish lid under darkfield conditions (0.63x, 2.5 optivar; Zeiss SteREO Discovery V8, Carl Zeiss Canada Ltd, Toronto, ON, Canada). Samples were taken from 4–6 plants at a time, and all tissue dissections and imaging occurred within a 2 hour timeframe. Complete sampling of an experimental series occurred between 8h30–22h30 on Day 1, and between 8h30–12h30 on Day 2. Under low nutrients, sampling began on 14 Aug 2021, 14 May 2022, and 16 Jun 2022. Under recommended nutrients, sampling began on 9 Oct 2021, 21 Apr 2022, and 3 May 2022.

ImageJ (https://imagej.nih.gov/ij/download.html) was used to determine organ surface area through manual selection of visible surface and calculated using the ROI manager. Stalked trichomes were manually counted using the Cell Counter plugin, allowing calculation of stalked trichomes per mm^2^.

### 2.4 Quantification of cannabinoid contents

A random sampling of dried inflorescences collected post-harvest from three of the four plants per inoculation treatment for a single nutrient regime experimental series were ground, separately, to fine homogeneous powders following freeze drying using a lyophilizer (SNL216V freeze-dryer, Thermo Savant Co. Ltd. USA). For each replicate, 0.2 g of sample was mixed with 20 mL of 100% ethanol in a 50 mL centrifuge tube. Tubes were placed on their side and shaken on a rotator for 5 min. One mL of the extract was transferred to an Eppendorf tube and centrifuged at 12,000 rpm for 5 min. Solvent was transferred to a 2 mL vial, either at the original concentration or 20x diluted in 100% ethanol.

Nine commercially available standards (purity > 98%) for cannabigerolic acid (CBGA), tetrahydrocannabinolic acid (THCA), cannabidiolic acid (CBDA), Δ^9^-tetrahydrocannabinol (Δ^9^-THC), cannabidiol (CBD), cannabichromene (CBC), cannabigerol (CBG), cannabinol (CBN), and Δ^8^-tetrahydrocannabinol (Δ^8^-THC) were obtained from Cerilliant (Round Rock, Texas, USA). Cannabinoid analysis was performed using the Agilent 1290 Infinity Ultra High-Performance Liquid Chromatography (UHPLC) system with an UV DAD detector (Agilent Technologies Inc., Santa Clara, CA, USA) set at 220 nm, for identification and quantification of the nine compounds, as per Lyu et al. (2022b). Due to sample overloading, CBDA and THCA required analysis at 20x dilution. All other cannabinoids were analyzed at their original concentrations.

### 2.5 Scanning electron microscopy

Individual calyces and bracts were submerged in two mL of 3.5% v/v formaldehyde in 0.025 M PIPES buffer. Samples were rotated overnight, followed by three rinses with 0.025 M PIPES buffer. Samples underwent ethanol ascensions of 30, 50, 70, 80, 95, and 100% for 30 min each, with three additional 100% ethanol rinses. Next, samples were critical-point dried with solvent-substituted CO_2_ (Leica EM CPD300, Leica Microsystems, Concord, ON, Canada). Samples were mounted on aluminum stubs with carbon mounts and rotary coated with 4 nm gold layer (Leica EM ACE200, Leica Microsystems, Concord, Canada). Samples were imaged under vacuum with a Hitachi TM-1000 scanning electron microscope operated at 15 kV (Hitachi Ltd., Chiyoda City, Japan).

### 2.6 Statistical analysis

Data analysis was performed using the statistical program SAS OnDemand for Academics, Enterprise Guide 8.3 for trichome data analysis (SAS Institute Inc. Cary, NC, USA). Version 9.4 of SAS OnDemand for Academics, Enterprise Guide was used for cannabinoid analysis (SAS Institute Inc. Cary, NC, USA). Differences in trichome densities between treatments and organs were evaluated using PROC GLM Tukey’s studentized range with the Dunnett adjustment for multiple comparisons, using a nested model. The level of significance was set at *p* < 0.05. Analysis of cannabinoid content was done using PROC GLIMMIX, using an interaction model with the same level of significance. **Table 3** provides the ANOVA results of the models for the low nutrient and recommended nutrient trichome datasets, both separately and together. This was evaluated over the course of May to June 2022.

**Table 3:** ANOVA values of the statistical models of the nutrient regimes.

Statistical Reporting of ANOVA for Trichome Densities under Nutrient Regimes				
	Type III Sums of Square	Mean Square	F-value	P-value
Low Nutrient Model	40216.3	5027.0	362.2	<0.0001
Recommended Nutrient Model	30801.3	3850.2	217.2	<0.0001
Nutrient regimes grouped together	70550.2	8818.8	523.7	<0.0001

For the control and consortium treatment for the recommended nutrient regime, n was 44 whereas for *Pseudomonas* sp. and *Bacillus* sp. treatments n was 48. n was 48 for all four treatments under the low nutrient regime. This discrepancy is due to 1 plant dying in each of the control and consortium treatments under the recommended nutrient regime, preventing trichome data collection and reducing the n value for their respective groups.

## 3 Results

### 3.1 Effect of PGPR inoculation on cannabis stalked trichome density

**Figure 2:**
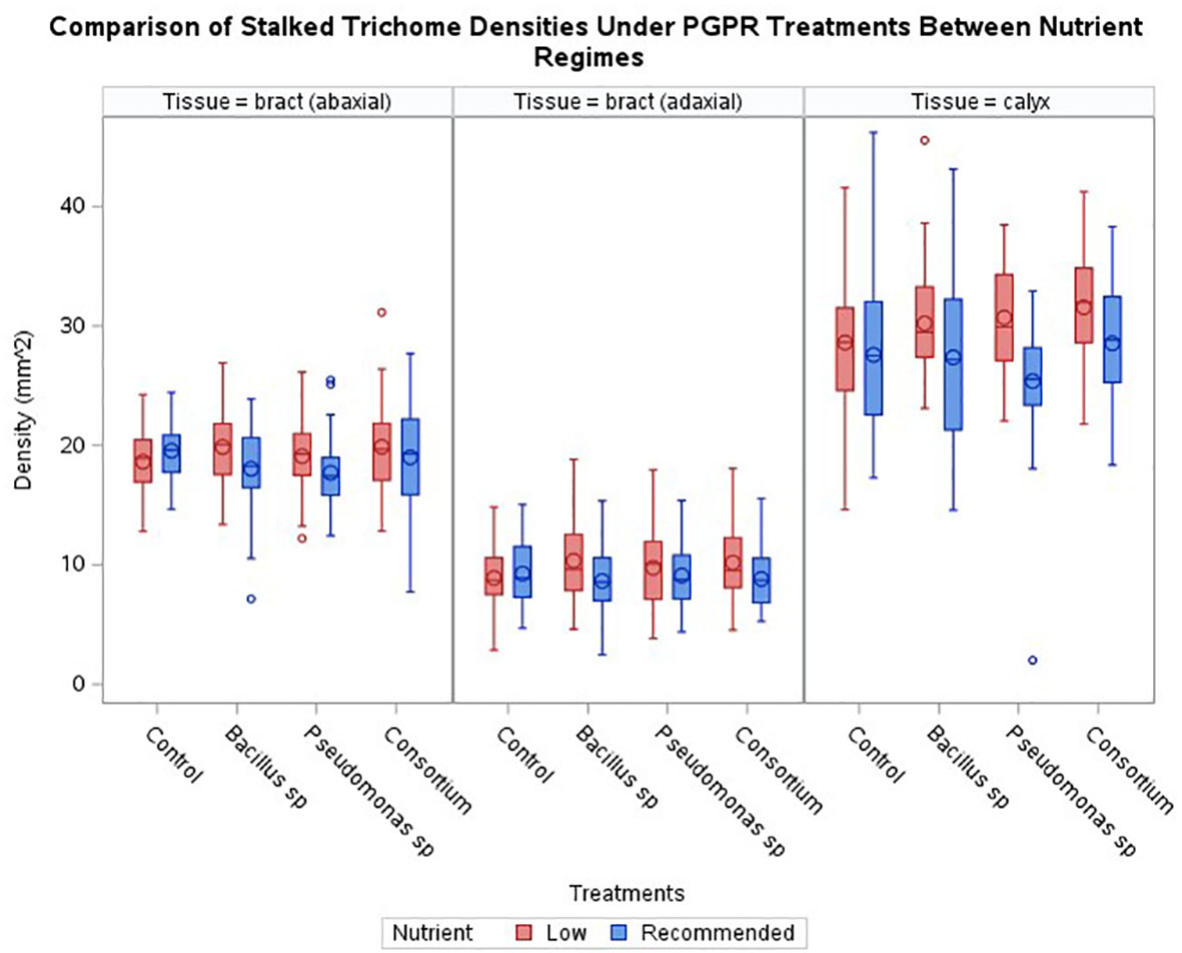
Comparisons of trichome densities by tissue and treatments under either recommended or low nutrients. No treatment had a statistically significant effect on enhancing trichome densities across cannabis inflorescence tissues under recommended nutrients, however two treatments had a statistically significant effect on enhancing trichome densities, one for *Bacillus* sp and one with consortium, under low nutrients. Horizontal bar within box indicates median value, circle within boxes indicates the mean. Vertical bars indicate data range. ‘o’ indicates outlier data points.

Across the three experimental series, generally, no increase in stalked trichome densities resulting from the bacterial treatments was observed when compared to the control (**Figure 2**). Only plants treated with *Pseudomonas* sp. displayed a statistically significant effect (*p* < 0.024) for the abaxial bract epidermis with a decrease in trichome density by 9.4%. Although not statistically significant, trichome densities tended to be slightly reduced in almost all other PGPR treated samples, except for the calyx of plants treated with the consortium inoculum, where a slight increase in trichome density of 3.5% was observed (*p* > 0.05).

### 3.2 PGPR inoculation coupled with a low nutrient regime affects stalked trichome density

Under the low nutrient regime, the general trend showed an increase in average stalked trichome densities on plants inoculated with PGPR compared to the recommended nutrient conditions (**Figure 2**). Within this trend, only the increase observed on the calyces of plants inoculated with the consortium treatment were significant (*p* < 0.0081) in comparison to non-inoculated, low nutrient regime plants. The increase caused by the presence of PGPR in these inadequate nutrient-supply treated plants was 10.3% for the consortium-treated calyx tissue compared to the control treatment for the low nutrient regime. The average densities of the other epidermal tissues under the PGPR treatments were not significant against the non-inoculated control.

**Figure 3:**
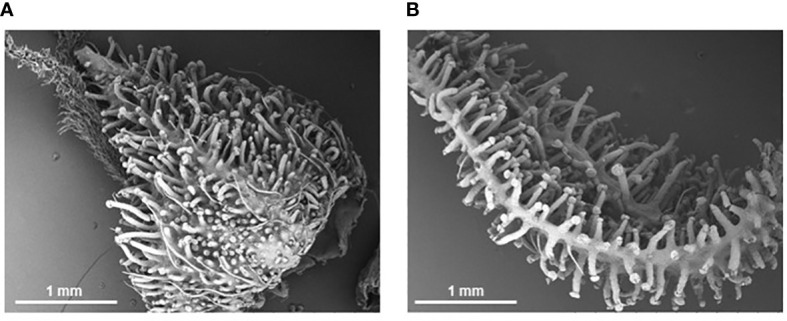
Scanning electron micrographs of cannabis inflorescence organs at Week 8 of flowering. Stalked trichomes completely cover the calyx **(A)** and bract **(B)** surfaces at time of harvest despite being under a low nutrient regime for the first half of development.

When comparing the trichome densities between the two nutrient regimes coupled with PGPR inoculations, an absence of bacteria under the low nutrient regime was the only treatment to cause a decrease in trichome densities compared to the recommended condition counterpart; a decrease of 4.7% for bract abaxial tissue and 4.0% for bract adaxial tissue, though an increase of 3.7% for calyx tissue was observed (**Figure 2**). Nonetheless, the administration of PGPR showed a trend to higher average trichome densities under low nutrient conditions than the level observed for the recommended nutrients under the same PGPR treatments (**Figure 2**). This is illustrated by **Figure 3**, where inflorescence epidermal tissue from the low nutrient regime remains covered by stalked trichomes. Comparing the changes in trichome densities between nutrient regimes, plants inoculated with the *Bacillus* sp. treatment showed the most consistent increase in stalked trichome densities on both organs under low nutrient conditions (**Figure 2**). Trichome densities on the bract abaxial, bract adaxial, and calyx epidermal tissues from low nutrient plants inoculated with *Bacillus* sp. increased by 10.1, 19.7, and 10.4%, respectively, compared to the recommended nutrient *Bacillus* sp. treatment group. For plants under *Pseudomonas* sp. inoculation, the low nutrient regime led to bract abaxial, bract adaxial, and calyx trichome densities increasing by 7.8, 7.0, and 21.0%, respectively, against their recommended nutrient counterpart. For plants treated with the consortium inoculum in the low nutrient regime, trichome densities on bract abaxial, bract adaxial, and calyx increased by 4.6, 15.5, and 10.6%, respectively, compared to consortium-inoculated plants grown under the recommended nutrients.

### 3.3 Cannabinoid content changes due to nutrient conditions and PGPR inoculations

**Figure 4:**
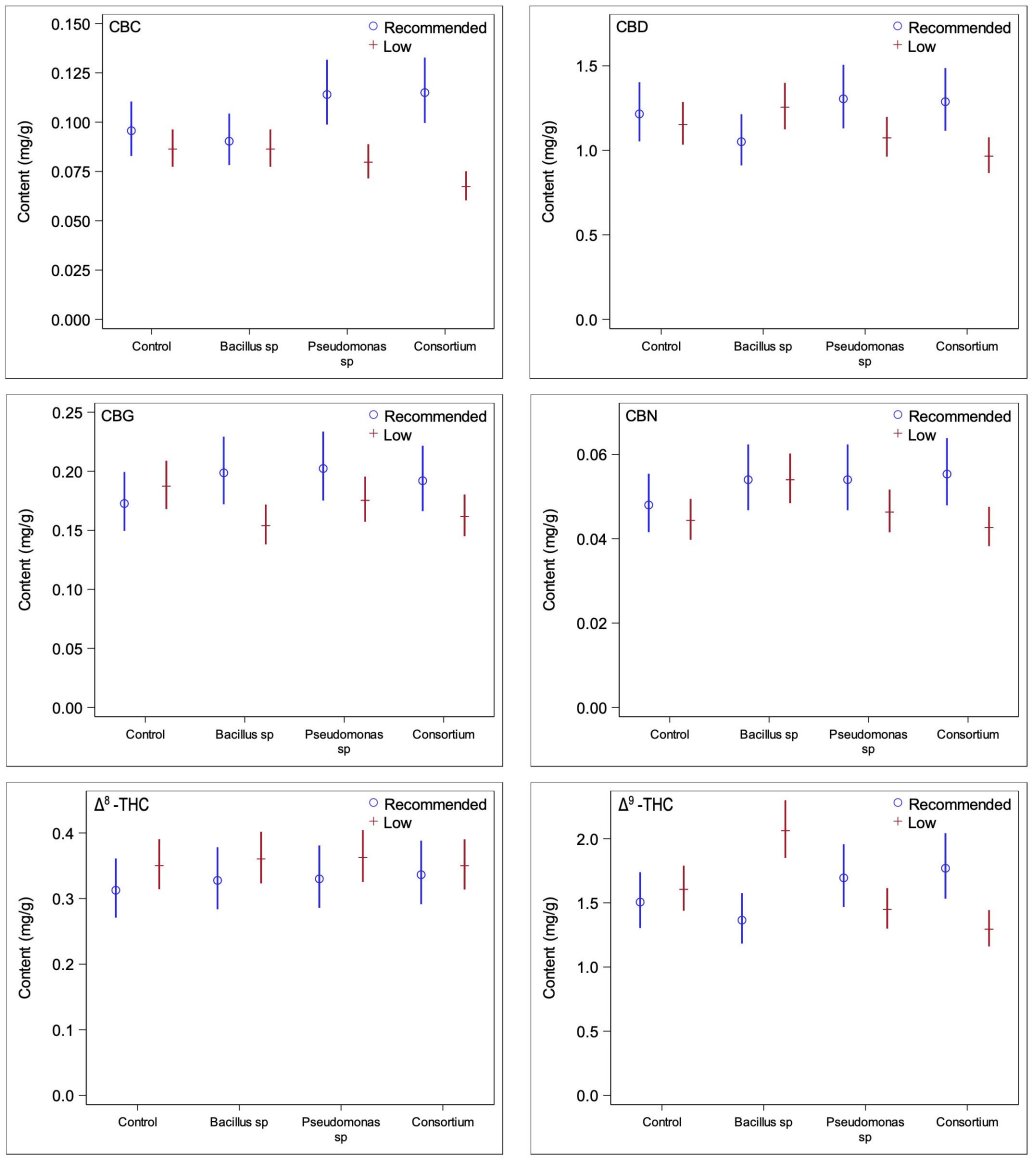
Comparisons of cannabinoid contents between low and recommended nutrient regimes across PGPR treatments. Under recommended nutrient conditions PGPR did not lead to any significant changes in cannabinoid content, whereas under low nutrient conditions, significant changes were observed for CBG and Δ^9^-THC under *Bacillus* sp. treatment and CBC under consortium treatment.

**Figure 5:**
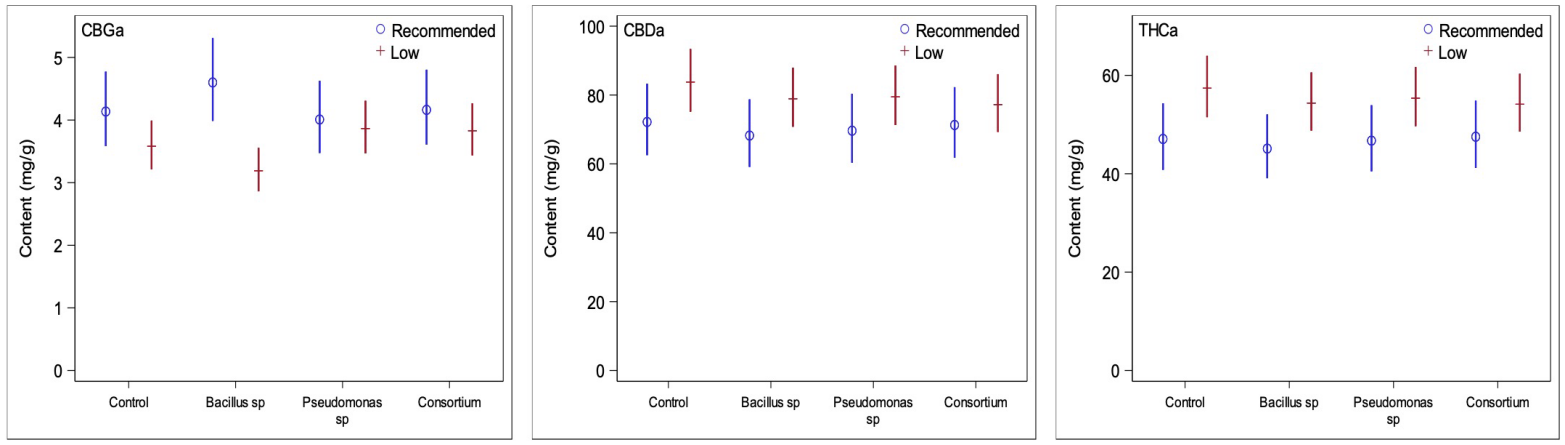
Comparisons of the three primary cannabinoids of interest between low and recommended nutrient regimes. Low nutrient regime consistently led to higher concentrations of CBDA and THCA than under recommended nutrients regardless of PGPR treatment. CBGA content on the other side was slightly higher under recommended nutrient conditions. This is likely due to recommended nutrient regimes not converting CBGA to CBDA and THCA at the same rate of the low nutrient regimes at time of harvest.

In order to ascertain whether changes in trichome densities are linked to changes in cannabinoid production, quantifications of cannabinoid contents from ground inflorescences under PGPR treatments and both nutrient conditions were obtained. Under recommended nutrient conditions, none of the concentrations of the cannabinoids measured were found to be significantly different in plants inoculated with bacteria compared to plants without bacterial treatment (**Figure 4**); the test for the statistical interaction model (treatment*compound) under the optimal nutrient regime only was not statistically significant (*p =* 0.56). This was consistent with the results of the trichome densities, as no PGPR treatment led to significantly greater trichome densities across all three inflorescence epidermal surfaces. When considering the two primary cannabinoids of interest, CBDA and THCA, PGPR-treated plants were found to have somewhat lower concentrations than those treated without PGPR, consistent with the trichome density trends previously observed (**Figure 5**); none of the differences were statistically significant, however.

Under the low nutrient regime, plants treated with either the *Bacillus* sp. or the bacterial consortium treatments featured significant changes in the concentrations of three cannabinoids each (**Figure 4**) (statistical interaction model *p* = <0.0001). The effect of *Bacillus* sp. was significant for CBG (*p* = 0.026), CBN (*p* = 0.022), and Δ^9^-THC (0.0019), with CBG decreased by 21.6%, CBN increased by 17.9%, and Δ^9^-THC increased by 22.2%. When inoculated with the bacterial consortium the CBC (*p* = 0.0008), CBD (*p* = 0.029), and Δ^9^-THC (*p* = 0.0048) cannabinoids were altered in significant quantities, with CBC decreased by 28.2%, CBD decreased by 19.4%, and Δ^9^-THC decreased by 24.0%.

The primary cannabinoid compounds of interest, CBDA, THCA, and CBGA, are also those that are the most abundant in cannabis inflorescence tissue. Under the low nutrient regime, the contents of CBDA and THCA were greater than under recommended conditions but were reduced for CBGA across all treatment groups (**Figure 5**), as expected as CBGA is the precursor molecule for THCA and CBDA. In the absence of PGPR, the low nutrient group showed an increase by 6.1% for CBDA, increase by 21.9% for THCA, and a decrease by 13.4% for CBGA, when compared to the non-inoculated control under recommended nutrient levels. The PGPR treatments caused increases of 15.6, 14.1 and 8.3% for CBDA, 20.5, 18.5 and 13.9% for THCA, and reductions by 30.7, 3.6 and 8.02% for CBGA for the *Bacillus* sp., *Pseudomonas* sp. and the consortium treatments, respectively. Interestingly, despite the low nutrient non-inoculated plants being the only group to have lower average trichome densities than its recommended nutrient counterpart, it yielded some of the greatest differences for the three cannabinoids of interest (**Figure 5**).

When comparing the total tested cannabinoids between the two nutrient regimes under the same PGPR treatments, the differences appear to be related to the presence of *Bacillus* sp. Referring to **Table 4**, the differences between the two nutrient regimes for four of the nine cannabinoids tested were significant in plants treated with *Bacillus* sp., of which the low nutrient regime was higher for all except for CBGA and CBG. Plants inoculated with the consortium treatment led to the same number of significant differences, with the levels of four cannabinoids being altered, however these were at lower concentrations than under the recommended regime. Plants with *Pseudomonas* sp. treatment only had changes in the concentrations of two cannabinoids (*p* < 0.05), CBC and CBD, both of which were reduced compared to the recommended regime. Lastly, in plants grown in the absence of PGPR, only THCA was affected (*p* < 0.05) by the nutrient regime, with its content being higher under low nutrients than recommended nutrient conditions.

**Table 4:** Statistically significant differences in cannabinoid content between low and recommended nutrient regimes of inoculated cannabis cv. CBD Kush.

Treatment	Statistically Significant Cannabinoids	% Change of Low Nutrient to Recommended Nutrient	Upper Limit	Lower Limit	P Values
Control	Tetrahydrocannabinolic acid	21.9%	0.02	0.38	0.031
*Bacillus* sp.	Cannabigerol	-22.5%	-0.43	-0.07	0.0058
	Cannabigerolic acid	-30.7%	-0.54	-0.19	<0.0001
	Tetrahydrocannabinolic acid	20.5%	0.007	0.37	0.042
	Δ^9^-Tetrahydrocannabinol	51.0%	0.23	0.59	<0.0001
*Pseudomonas* sp.	Cannabichromene	-30.1%	-0.54	-0.18	0.0001
	Cannabidiol	-17.7%	-0.37	-0.01	0.034
Consortium	Cannabichromene	-41.4%	-0.71	-0.35	<0.0001
	Cannabidiol	-25.0%	-0.47	-0.12	0.0019
	Cannabinol	-22.9%	-0.44	-0.08	0.0049
	Δ^9^-Tetrahydrocannabinol	-26.9%	-0.49	-0.13	0.0008

Positive values indicate an increase when under low nutrient conditions, and a negative value indicates a decrease when under low nutrients, in comparison to the recommended nutrient regime.

## 4 Discussion

This study has demonstrated that while PGPR inoculations do not have a significant effect on stalked trichome densities on cannabis inflorescence organs when plants were grown under recommended nutrient conditions, the application of an environmental stress for the first half of plant development reveals a benefit to applying these microbes. Under recommended nutrient conditions, there was a surprising downward trend of trichome densities in relation to the addition of PGPR on all three evaluated epidermal tissues, but this trend was reversed under the low nutrient regime. Though non-inoculated plants treated with the low nutrient regime manifested a decrease in trichome densities compared with the recommended nutrient regime across all epidermal types, PGPR rescued this effect on trichome numbers. This is consistent with the limited studies available that have provided links to trichome densities with PGPR presence in *Ocimum basilicum* (Copetta et al., 2006) and environmental stressors in *Cymbopogon citratus* (Mirzaie et al., 2020). However, not only were these studies conducted on different plant species, but the stress was related to the effect of drought only. This makes ours an early-stage investigation to determine both if PGPR presence affect trichome quantities on cannabis plants and the impact of nutrient stress on trichome densities, but also how these two factors influence trichome development when simultaneously administered.

The true benefit of manipulating cannabis growth conditions lies in the application of an environmental stress, revealed by the cannabinoid contents. Under the recommended nutrient conditions, PGPR did not cause meaningful differences in the abundance of minor cannabinoids. The abundance of two cannabinoids of commercial interest, CBDA and THCA, was reduced by the PGPR but under the low nutrient regime PGPR increased their contents. For example, *Bacillus* sp. inoculation under the low nutrient regime increased the Δ^9^-THC content, a degradation product of THCA, by 51% against the amount detected in the recommended nutrient regime’s *Bacillus* sp. treatment. This effect of low nutrient stress is consistent with Caplan et al. (2017), who investigated the link between substrate and liquid fertilizer application rates. Caplan et al. (2017) found that while a lower fertilizer rate led to reduced yield, there were higher cannabinoid concentrations with a particular substrate. As we have shown, this link is marginally reflected in enhanced trichome densities, but seems to be more evident of an increased level of cannabinoid biosynthesis per trichome. What was most surprising, however, was all bacterial inoculation treatments increasing the amount of THCA and CBDA in the low nutrient regimes compared to the recommended nutrient counterparts. It should be noted that the reduction in CBGA under low nutrient conditions compared to recommended conditions is likely due to it being the precursor molecule to THCA and CBDA, and as these were present in higher amounts under low nutrient regimes; it is consistent that CBGA content would be lower than that of the recommended nutrient regime treatments. The observations on cannabinoid concentrations differ somewhat from previous work from our laboratory (Lyu et al., 2022b), an effect that is likely due to the type of rooting medium used. Plants in the present study were grown in soil with coconut fibre, which is recommended by the manufacturer for cannabis and cutting propagation, whereas Lyu et al. used soil with compost which provides additional beneficial microorganism and nutrient sources; this could have led to the differences in cannabinoid profiles between treatments. Clearly much remains to be learned in this regard, however as both Bevan et al. (2021) and Caplan et al. (2017) demonstrated, cannabis can have unique responses to tailored growth conditions and environments; future studies may be guided based on comparing how the effects of microbial inoculations may change for cannabis based on the rooting medium used. Notably, in cannabis production, an increased ‘yield’ is not necessarily equated with an increase in biomass, but ‘yield’ may instead represent an increase in cannabinoid concentration per biomass, possibly at the cost of reduced overall biomass. Only experiments conducted at larger scale will reveal whether this can be translated into higher cannabinoid yield per production surface. Secondly, ‘yield’ may relate to a particular cannabinoid rather than the overall combined amount and may pertain to a desired ratio between certain compounds rather than their absolute abundance. Growth conditions must therefore be tailored to the desired outcome which may require a carefully formulated combination of growth-enhancing and growth-stressing conditions. As the current Canadian cannabis market demands increasingly higher THC contents, it is imperative that producers have clear science-based guidance available in this regard in order to make informed decisions about their growing strategies.

This study has provided evidence to justify the incorporation of eco-friendly growth conditions into indoor cannabis production under nutrient-stressed conditions. While the PGPR treatments had marginal effects on the trichome densities and did not necessarily enhance effects on cannabinoid contents, it was the reduction in the amount of applied nutrients for the first half of plant development that led to a noticeable enhancement in the primary cannabinoids of interest, namely THC and CBD and their counterparts, particularly when inoculated with *Bacillus* sp. This leads us to potentially recommend the practice of restricting nutrient applications for cannabis plants, and while in general the presence of PGPR only yielded moderate changes in trichome count and cannabinoid profile, the addition of *Bacillus* sp. correlated to the greatest number of changes in cannabinoid profiles between recommended and low nutrient regimes. Future work should investigate the economic potential of these results for producers with regards to yield versus production costs, as well as the extent of reduced environmental stress stemmed from both reducing the amount of nutrients manufactured and diminishing the concentration of contaminated wastewater leaving facilities. Overall, this study not only demonstrated that PGPR inoculation has a limited impact on cannabis stalked glandular trichomes, but notably how the application of an environmental stressor can elicit improved effects of these inoculations, thereby motivating changes towards production methods that minimize chemical inputs.

## Data availability statement

The raw data supporting the conclusions of this article will be made available by the authors, without undue reservation.

## Author contributions

CT and DS designed the experiments. CT performed the experiments. DL and ER provided the cannabinoid data. CT, TS, DS and AG analyzed the data. CT wrote the manuscript. DS and AG edited the manuscript and provided feedback. All authors contributed to the article and approved the submitted version.
